# Coding Deficits in Noise-Induced Hidden Hearing Loss May Stem from Incomplete Repair of Ribbon Synapses in the Cochlea

**DOI:** 10.3389/fnins.2016.00231

**Published:** 2016-05-25

**Authors:** Lijuan Shi, Yin Chang, Xiaowei Li, Steven J. Aiken, Lijie Liu, Jian Wang

**Affiliations:** ^1^Department of Physiology, Medical College of Southeast UniversityNanjing, China; ^2^School of Human Communication Disorders, Dalhousie UniversityHalifax, NS, Canada

**Keywords:** auditory coding, auditory nerve fibers, ribbon synapse, noise, hidden hearing loss

## Abstract

Recent evidence has shown that noise-induced damage to the synapse between inner hair cells (IHCs) and type I afferent auditory nerve fibers (ANFs) may occur in the absence of permanent threshold shift (PTS), and that synapses connecting IHCs with low spontaneous rate (SR) ANFs are disproportionately affected. Due to the functional importance of low-SR ANF units for temporal processing and signal coding in noisy backgrounds, deficits in cochlear coding associated with noise-induced damage may result in significant difficulties with temporal processing and hearing in noise (i.e., “hidden hearing loss”). However, significant noise-induced coding deficits have not been reported at the single unit level following the loss of low-SR units. We have found evidence to suggest that some aspects of neural coding are not significantly changed with the initial loss of low-SR ANFs, and that further coding deficits arise in association with the subsequent reestablishment of the synapses. This suggests that synaptopathy in hidden hearing loss may be the result of insufficient repair of disrupted synapses, and not simply due to the loss of low-SR units. These coding deficits include decreases in driven spike rate for intensity coding as well as several aspects of temporal coding: spike latency, peak-to-sustained spike ratio and the recovery of spike rate as a function of click-interval.

## Noise induced synaptic damage

Noise-induced hearing loss has traditionally been quantified by changes in auditory sensitivity evidenced by shifts in hearing threshold (Borg et al., 1995). Physiologically, the loss of auditory sensitivity after noise exposure is largely due to changes in the functional status of outer hair cells (OHCs) in the cochlea, which provide mechanical amplification for soft sounds (Hudspeth, 1997; Szalai et al., 2011). Noise exposures at some levels and durations can impact OHC function to produce temporary threshold shifts (TTS) with no evidence of OHC death. In such cases, the recovery of OHC function is evidenced by recovery of otoacoustic emissions (OAE; Subramaniam et al., 1994; Chang and Norton, 1996; Kujawa and Liberman, 2009) and cochlear microphonics (CM; Wang et al., 1992, 2011; Chen et al., 1995; Chen and Liu, 2005; Chen and Zhao, 2007) in parallel with the recovery of thresholds, as well as by the repair of related structures such as the stereocilia and the tectorial membrane (Wang et al., 2002, 2011). In contrast, noise exposures at higher levels and/or for longer durations can cause permanent damage or death of OHCs, and result in PTS. Therefore, the OHC and surrounding structures are considered to be the major loci of cochlear damage underlying noise-induced threshold shifts (Sohmer, 1997; Henderson and Subramaniam, 2011).

While the inner hair cells (IHCs) are much less likely to be killed by noise, it has long been recognized that the synapses between IHCs and auditory nerve fibers (ANFs) are sensitive to noise (Spoendlin, 1971; Liberman and Mulroy, 1982; Robertson, 1983). In early studies, noise-related synaptic damage was mainly attributed to damage to post-synaptic terminals, while more recent studies have also found damage to pre-synaptic ribbons (Kujawa and Liberman, 2009).

### Is noise-induced synaptic damage reversible?

There is controversy as to whether noise-damaged synapses can be repaired, but partial recovery of synaptic connections between IHCs and ANFs after initial disruption by noise has been reported in a number of studies (Pujol et al., 1993; Puel et al., 1997, 2002; Pujol and Puel, 1999). The recovery of synaptic damage has been counted as evidence for its role in TTS (Mulroy et al., 1990; Henry and Mulroy, 1995), although it is almost impossible to differentiate the impact of synaptic damage from OHC damage on TTS, and evidence for synaptic repair has drawn criticism due to its reliance on non-quantitative measures of “recovery.” More recently, permanent synaptic damage has been found in animal studies in which the numbers of synaptic ribbons and post-synaptic terminals were observed dynamically over a period of time after a noise exposure that did not cause PTS. In a pioneering study using CBA mice, the initial loss in synapses following noise exposure was consistent with the final loss of spiral ganglion neurons (SGNs) tested 2 years later, suggesting that the lost synapses were not recovered (Kujawa and Liberman, 2009). However, a study in guinea pigs conducted by the same team found a similar initial loss of ribbon synapses (Lin et al., 2011), but much smaller final loss of SGNs. This suggests that some SGNs that originally lost their synapses with IHCs survived because of a re-establishment or repair of the synapse. In two of our previous papers, we also found a significant recovery of synapse counts in guinea pigs in the month following a non PTS-inducing noise exposure (Liu et al., 2012; Shi et al., 2013). Given the reported differences between mice and guinea pigs as summarized above, there may be a species difference in the ability to regenerate synapses after noise-induced damage. However, another recent study in C57 mice reported a reversible loss of ribbon synapses after a similar non PTS-inducing noise (Shi et al., 2015). This discrepancy calls for further investigation.

**Figure 1:**
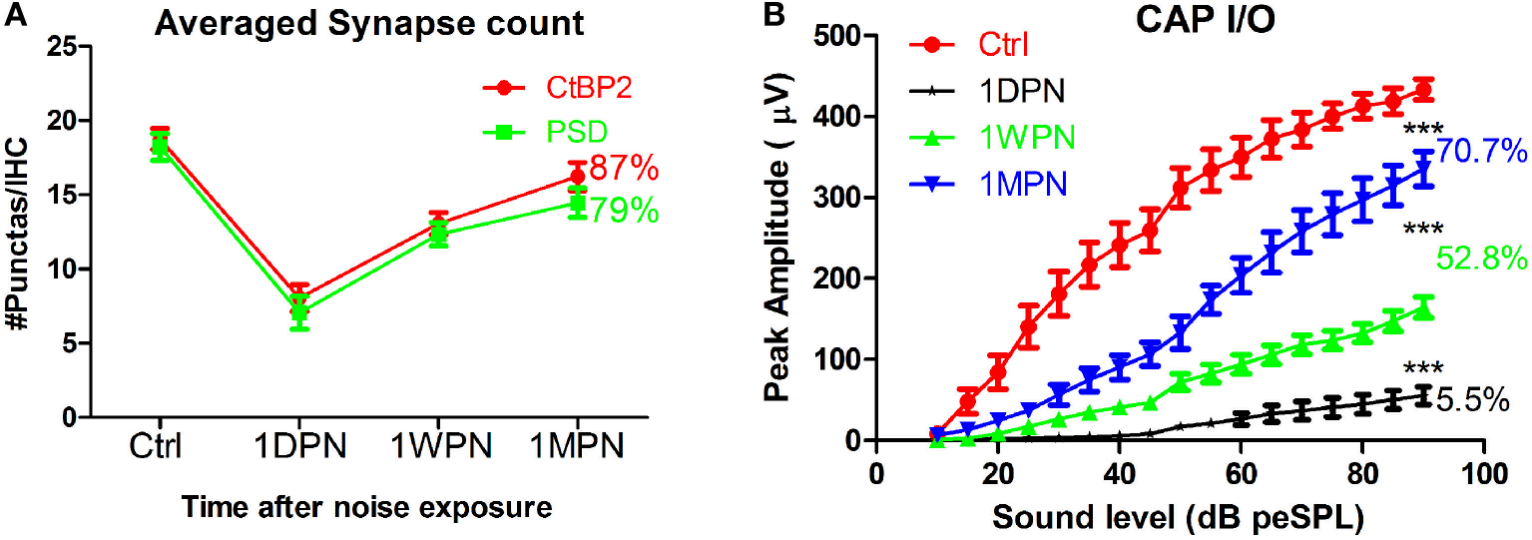
**Changes of synapse counts (A) and input/output functions of compound action potentials by click (B)**. Synapses were stained by antibodies for C-terminal binding protein 2 (CtBP2) and post-synaptic density 95. One-way ANOVA was performed for CAP amplitude at 90 dB peSPL (peak equivalent sound pressure level). The number of percentages was calculated against the control (^***^p < 0.001).

In a recent review (Kujawa and Liberman, 2015), the synaptic recovery found in our guinea pig studies was disputed and attributed to up/down regulation of synaptic proteins measured in immunohistology rather than re-generation of the synaptic connection. However, several lines of evidence support the possibility of synapse repair following noise-induced damage. Firstly, studies have shown plastic changes in the pre-synaptic component (Ruel et al., 2007), including the size and location of ribbons (Shi et al., 2013). Secondly, changes in the amplitude of the click-evoked compound action potential (CAP), recorded via a round window electrode, correspond very well with measured changes in synapse counts (Figure 1). In our data, CAP amplitude was reduced to ~5% after noise exposure (1DPN; at the maximum sound level), corresponding to a massive disruption of synapses and reduced function of surviving synapses. However, CAP amplitude had partially recovered at 1 week and 1 month post noise exposure (1WPN and 1MPN, 52.8 and 70.7% of the control amplitude, respectively). This could not occur without a partial re-establishment of the synapses that were initially disrupted. A one-way ANOVA found a significant effect of noise exposure (*F*_3_ = 128.9, *p* < 0.001) on CAP amplitude, and *post-hoc* pairwise comparisons (Holm-Sidak) showed significant but incomplete recovery of amplitude in agreement with the change in synapse counts. It is therefore reasonable to conclude that noise-induced synaptic disruption is, at least partially, repairable.

## Synapse damage and hidden hearing loss

The synapses between IHCs and type I ANFs are characterized as having pre-synaptic dense bodies called ribbons (Merchan-Perez and Liberman, 1996; Fuchs, 2005; Tom Dieck et al., 2005; Nouvian et al., 2006; Bulankina and Moser, 2012). The functional roles of ribbons in synaptic transmission has been associated with their ability to tether synaptic vesicles in close proximity to the neurotransmitter release sites called active zones (Moser et al., 2006; Nemzou et al., 2006; Buran et al., 2010; Uthaiah and Hudspeth, 2010). Animals with deletion of a major part of the Bassoon gene (Altrock et al., 2003) lack synapse-anchored ribbons at most IHC active zones and this is associated with a huge reduction in the readily releasable pool of synaptic vesicles (Khimich et al., 2005; Schnee et al., 2005; Tom Dieck and Brandstätter, 2006). This mutation results in a significant deficit in temporal coding ability (Moser et al., 2006, 2013; Buran et al., 2010), which is critical for auditory signal processing.

Following classic studies by Liberman and his colleagues (Kiang et al., 1982; Liberman, 1982), ANFs are functionally categorized by their spontaneous rates (SRs), which are consistently related to properties of their rate-level functions (firing rate vs. sound pressure level). Spontaneous rates are inversely related to both thresholds and dynamic ranges, i.e., low-SR fibers begin to respond at higher sound levels and continue to increase their firing rates over a larger dB range of sound levels than their high-SR counterparts (Liberman, 1978; Costalupes, 1985; Young and Barta, 1986). This is important for many reasons (Plack et al., 2014; Eggermont, 2015; Heil and Peterson, 2015; Kujawa and Liberman, 2015). For example, low-SR units are considered to be critical for hearing in noisy environments due to their larger dynamic response ranges, wider distribution of thresholds, and their ability to follow the time envelope of signals. In contrast, high-SR units are highly sensitive to soft sounds and are saturated by high-level background noise (Costalupes, 1985; Young and Barta, 1986; Plack et al., 2014; Eggermont, 2015; Heil and Peterson, 2015).

Damage to ribbon synapses in the absence of permanent threshold shifts should impact function, but since this cannot be detected via standard audiometric assessment, this has been called “hidden hearing loss.” The precise functional deficits in such cases remain to be determined, but a recent finding suggests that the damage might be selective with respect to SR. A selective loss of low-SR ANFs was found after a non PTS-inducing noise exposure (Furman et al., 2013)—currently the only study reporting single unit data following non PTS-inducing noise exposure. The loss of low-SR units was likely secondary to damage to the respective synapses. This is significant because low-SR fibers are thought to be critical for signal coding in noisy backgrounds (Costalupes, 1985; Young and Barta, 1986; Furman et al., 2013; Plack et al., 2014; Kujawa and Liberman, 2015; Liberman et al., 2015). Selective low-SR damage could be an important feature of noise-induced hidden hearing loss (Plack et al., 2014), but the impact on signal coding has yet to be documented. In the above mentioned paper, no significant auditory coding differences were found between control and noise-exposed animals.

### Coding deficits developed with synaptic repair

**Figure 2:**
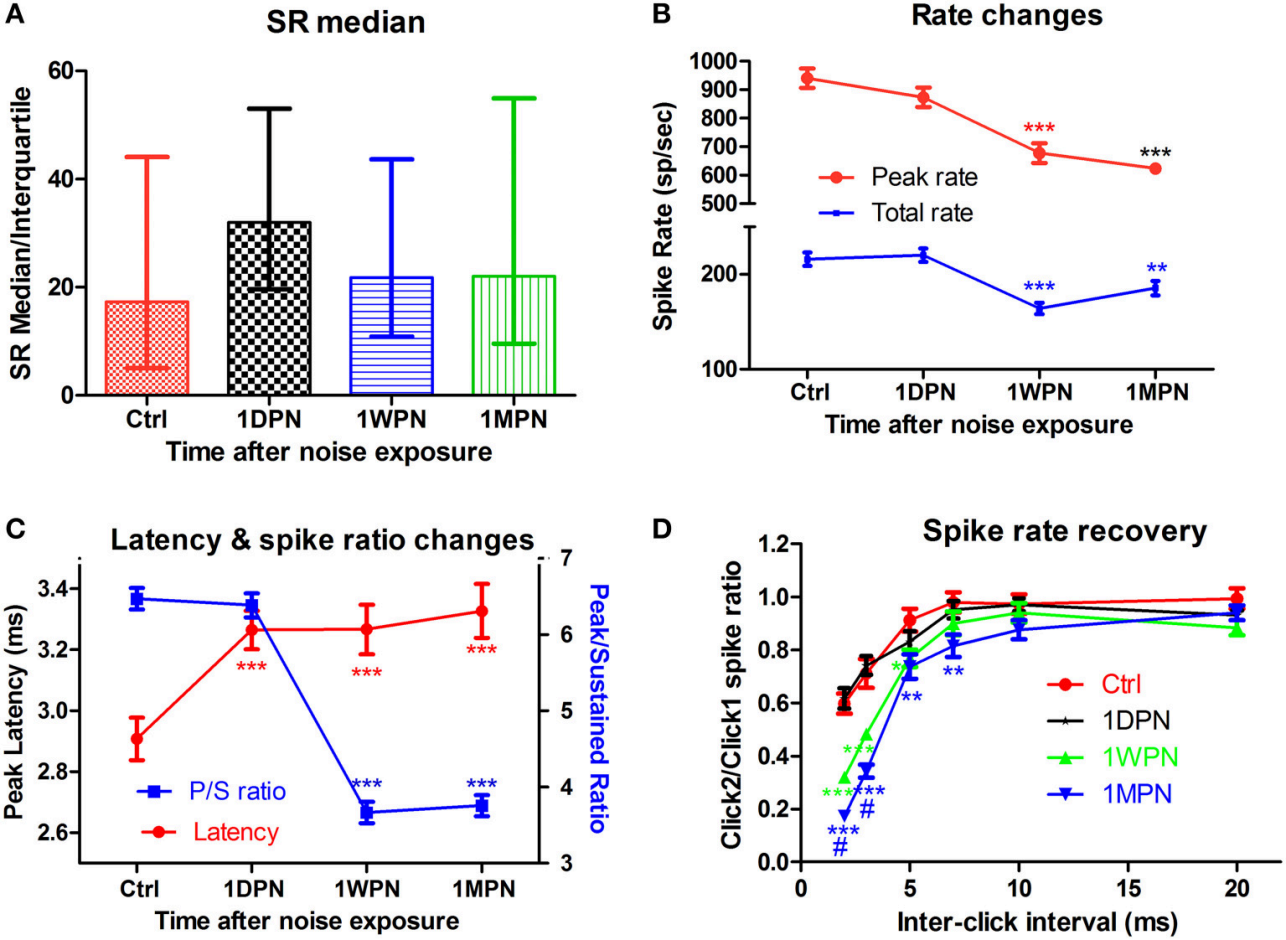
**Impact of noise on single unit activity of ANFs. (A)** The transient change of SR in high frequency region (beyond 4 kHz). **(B)** Decrease in both peak and total spike rate. **(C)** Prolonged peak latency and decreased peak/sustained spike ratio. **(D)** Normalized recovery of spike rate evoked by the 2nd click as a function of inter click interval, showing delayed recovery in damaged cochleae. Tests in **(B–D)** are all from low-SR units (^**^p < 0.01 and ^***^p < 0.001, respectively, for comparisons against Ctrl; ^#^*p* < 0.05 against 1WPN).

In contrast to the (single time-point) findings reported by (Furman et al., 2013). We found that the noise-induced alteration in SR distribution was transient. In our study, ~200 units were recorded from control and experimental animals tested at three time points after an exposure to a non PTS-inducing noise (broadband noise at 105 dB SPL for 2 h). A large initial change in the ratio of low- to high-SR units was measured in the high frequency region (best frequency > 4 kHz) at 1DPN, but this largely recovered. The ratio in controls was 1.2 (55 low-SR units vs. 47 high-SR units with SR cutoff at 20 spikes/s), which fell to 0.35 at 1DPN (18/52) and recovered to 0.89 at 1WPN (43/51) and 1.09 (47/43) at 1MPN (Figure 2A). This was verified by a one-way rank ANOVA (Kruskal-Wallis) which showed a significant effect of noise exposure (*H*_3_ = 13.314, *p* = 0.001). Most importantly, a significant difference in the ratio of low- to high-SR units was only found between control units and those obtained at 1DPN (Tukey *post-hoc* analysis, *Q* = 5.551, *P* < 0.05).

Coding deficits are summarized in Figure 2 for low-SR units in the high-frequency region because these were the only ANFs in the high frequency region showing significant changes. ANF intensity coding is based on two associated mechanisms: spike rate and phase locking changes (volley principle) as a function of sound level (Heil and Peterson, 2015). In the present study, our observations were limited to spike rate changes, which were recorded at a fixed sound level and compared across groups to show the effects of noise induced damage in the month following the noise exposure. ANOVAs showed a significant reduction in both peak and total spike rate at 1WPN and 1MPN (*F*_3_ = 22.86, *p* < 0.001 for peak rate and *F*_3_ = 14.03, *p* < 0.001 for total rate), but not immediately following the noise exposure (1DPN; Figure 2B). In combination with the change in the CAP (Figure 1B), the results suggest degraded ANF encoding of sound intensity.

Although ANF temporal coding is often evaluated by measuring phase-locking to the temporal envelope (Bharadwaj et al., 2014), changes in ANF onset responses have been used to demonstrate temporal coding deficits related to ribbon synapse mutations (Buran et al., 2010; Jing et al., 2013). In the present study, a deterioration in onset responses was evidenced by a decrease in peak rate (Figure 2B), an increase in peak latency (as shown by a one-way ANOVA, *F*_3_ = 7.061, *p* = 0.0001) and a decrease in the peak/sustained spike ratio (one-way ANOVA, *F*_3_ = 117.0, *p* < 0.001, Figure 2C). A deterioration in the click-evoked onset response was also found (Figure 2D) using a paired-click paradigm, with noise-exposed fibers requiring a longer inter-click interval for spike rate recovery in response to the second click. A two-way ANOVA of noise exposure vs. inter-click interval found a significant effect of noise exposure. *Post-hoc* pairwise tests found that second-click spike rates were significantly lower than controls in the 1 WPN and 1 MPN groups, but not at 1 DPN (see asterisks).

In summary, while the data showed a greater initial loss of synapses to low-SR ANF units, this did not cause significant coding changes in the ANF population as a whole. Surprisingly, coding deficits occurred later—at 1WPN and 1MPN—in the most of the tested coding behaviors. In light of the recovery of synapse counts at the two later time points, a reasonable interpretation of the functional data is that temporal coding deficits arose in the re-established synapses.

## Implications and future directions

The functional significance of hidden hearing loss needs to be comprehensively evaluated because noise causing such hearing loss may occur frequently in daily life (Ivory et al., 2014) and is generally considered to be safe according to current noise exposure safety standards. Although ribbon synapse damage is partially repairable, damage may accumulate across the lifespan and may contribute to perceptual difficulties commonly experienced in the elderly (Plack et al., 2014). However, the problem cannot be simply attributed to the selective loss of low-SR units. Rather, the SR distribution may largely recover via a process of synaptic repair, with coding deficits occurring as a result of incomplete repair.

In the future, ANF single unit behavior should be studied over a longer period to determine whether coding deficits found in the month following noise exposure are temporary or persistent. Furthermore, it will be important to investigate (1) why synapses innervating low-SR units are extremely sensitive to noise, and (2) why the repaired synapses are functionally abnormal. Related to (1), investigation is needed to explore the mechanisms involved in noise-induced damage to pre-synaptic ribbons, as these mechanisms are much more poorly understood than mechanisms involved in noise-induced damage to post-synaptic terminals.

## Materials and methods (for single unit study)

Albino guinea pigs with red eyes, irrespective of gender, were obtained for the single unit study from the Experimental Animal Service of Southeast University, a qualified provider for laboratory animals. In total, 64 guinea pigs aged 2–3 months were used and were divided into a control group (Ctrl: *n* = 16) and a noise exposure group (*n* = 48). The latter group was further divided into 3 subgroups according to the time of observation as 1 day, 1 week and 1 month post noise exposure (1DPN, 1WPN, and 1MPN; 16 in each subgroup). All animal procedures were approved by the University Committee for Laboratory Animals of Southeast University, China (Permit number: SYXK 2011-0009).

The animals in the noise group were exposed to a single dose of broadband noise at 105 dB SPL for 2 h when they were awake. They were unrestrained in a cage 60 cm below the horns of two loudspeakers; one was a low frequency woofer and the other was a high frequency tweeter. Electrical Gaussian noise was delivered to the speakers after power amplification. The acoustic spectrum of the sound was distributed mainly below 20 kHz as reported previously (Liu et al., 2012). The frequency range for sound density 10 dB below the peak was 3–14 kHz. The noise level was monitored using a Âĳ-inch microphone linked to a sound level meter (Microphone: 2520, SLM: 824, Larson Davis, Depew, NY, USA).

During the single unit recording, the animal was anesthetized initially by ketamine in combination with Rompon (40 + 10 mg/kg, respectively, i.p.) and maintained with pentobarbital (10 mg/kg/h). Body temperature was maintained at 38°C with a thermostatic heating pad. Trachea intubation was performed and respiration was artificially maintained during the single unit recording, which typically lasted 6–8 h. A post-neck approach was used to explore the trunk of auditory nerve. Glass micropipettes were used as electrodes, with impedances between 10 and 20 MW when filled with 1 M NaCl. The electrode was advanced remotely by a micro-positioner (Model 2650, David KOPF Instruments, Tujunga, CA, USA) when a broadband noise burst of 30 ms was presented at a level around 75 dB SPL. Neuronal responses were led from the electrode to the headstage of a microeletrode AC Amplifier (Model 1800, A-M Systems, Carlsborg, WA, USA). The output of the amplifier was sent to an RZ5 processor from Tucker-Davis Technologies (TDT system III, Alachua, FL, USA) for digitizing and further processing. Sound stimulation and recording were controlled by Brainware software via the TDT RZ6.

Once a neuron was isolated via a unit search (using a noise burst), the following measures were recorded: (1) spontaneous spikes over a 30 s interval, (2) unit threshold as estimated with noise bursts of various levels, (3) best frequency (BF) as estimated with an iso-intensity-frequency curve obtained by presenting 50-ms tone bursts of different frequencies at a level of 20 dB above the noise-burst threshold, and (4) peristimulus histogram in response to a 50-ms tone burst at BF and 20 dB above threshold. In test (3), the stimulus at each frequency was repeated 50 times, while in test (4) the stimulus was repeated 150 times. From test (4), peak latency, peak spike rate, adapted (sustained) spike rate as well as the peak/adapted spike rate ratio were measured or calculated.

### Statistical analysis

All data are expressed as means ± the standard error of the mean (SEM). Analysis of variance (ANOVA, one way or two way) was performed using SigmaPlot 12 software. *P* < 0.05 was used as the significance criterion for all tests. All statistical figures were made using Graphpad Prism 5.

## Author contributions

JW, research design, data collection and analysis, paper writing. LS, data collection and analysis. SA, data analysis and paper writing. LL, data collection, analysis and paper writing. YC, data collection and analysis.

### Conflict of interest statement

The authors declare that the research was conducted in the absence of any commercial or financial relationships that could be construed as a potential conflict of interest.

## Abbreviations

ANF: Auditory nerve fiber
CM: cochlear microphonice
CAP: compound action potential
IHC: Inner hair cell
OHC: outer hair cell
OAE: optoacoustic emissions
PTS: permanent threshold shift
SR: spontaneous rate
TTS: temporary threshold shift
SGNs: spiral ganglion neurons
1DPN: 1 day post noise
1WPN: 1 week post noise
1MPN: 1 month post noise.
